# Efficacy of Radiofrequency as Therapy and Diagnostic Support in the Management of Musculoskeletal Pain: A Systematic Review and Meta-Analysis

**DOI:** 10.3390/diagnostics12030600

**Published:** 2022-02-26

**Authors:** Giacomo Farì, Alessandro de Sire, Cettina Fallea, Mariantonia Albano, Gianluca Grossi, Elisa Bettoni, Stefano Di Paolo, Francesco Agostini, Andrea Bernetti, Filomena Puntillo, Carlo Mariconda

**Affiliations:** 1 Department of Basic Medical Sciences, Neuroscience and Sensory Organs, University of Bari, 70121 Bari, Italy; 2 Department of Medical and Surgical Sciences, University of Catanzaro “Magna Graecia”, 88100 Catanzaro, Italy; 3 Department of Rehabilitation Sciences, Humanitas Gradenigo Hospital, 10153 Turin, Italy; cettinafallea@gmail.com (C.F.); albanoalba@hotmail.it (M.A.); gianlu.grossi@outlook.it (G.G.); elisa.bettoni83@gmail.com (E.B.); carlo.mariconda@alice.it (C.M.); 4 Department of Biomedical and Neuromotor Science, IRCCS Rizzoli Orthopedic Institute, 40136 Bologna, Italy; stefano.dipaolo@ior.it; 5 Department of Anatomical and Histological Sciences, Legal Medicine and Orthopedics, Sapienza University, 00185 Rome, Italy; francescoagostini.ff@gmail.com (F.A.); andrea.bernetti@uniroma1.it (A.B.); 6 Department of Interdisciplinari Medicine, Aldo Moro University, 70124 Bari, Italy; filomena.puntillo@uniba.it

**Keywords:** radiofrequency, pain, rehabilitation, musculoskeletal disorders, osteoarthritis, interventional physiatry

## Abstract

Radiofrequency (RF) is a minimally invasive procedure used to interrupt or alter nociceptive pathways for treating musculoskeletal pain. It seems a useful tool to relieve chronic pain syndromes, even if, to date, solid evidence is still needed about the effectiveness of this therapy. By this systematic review and meta-analysis, we aimed to evaluate the efficacy of RF in treating musculoskeletal pain. PubMed, Medline, Cochrane, and PEDro databases were searched to identify randomized controlled trials (RCTs) presenting the following: patients with chronic musculoskeletal pain as participants; RF as intervention; placebo, anesthetic injection, corticosteroid injection, prolotherapy, conservative treatment, physiotherapy, and transcutaneous electrical nerve stimulation as comparisons; and pain and functioning as outcomes. Continuous random-effect models with standardized mean difference (SMD) were used to compare the clinical outcomes. Overall, 26 RCTs were eligible and included in the systematic review. All of them analyzed the efficacy of RF in four different regions: cervical and lumbar spine, knee, sacroiliac (SI) joint, shoulder. The outcomes measures were pain, disability, and quality of life. A medium and large effect in favor of the RF treatment group (SMD < 0) was found for the shoulder according to the Visual Analogical Scale and for the SI joint according to the Oswestry Disability Index. A small effect in favor of the RF treatment group (SMD > 0) was found for the spine according to the 36-item Short Form Survey. Non-significant SMD was found for the other outcomes. RF represents a promising therapy for the treatment of chronic musculoskeletal pain, especially when other approaches are ineffective or not practicable. Further studies are warranted to better deepen the effectiveness of RF for pain and joint function for each anatomical region of common application.

## 1. Introduction

Radiofrequency (RF) is a minimally invasive procedure that is widely used for treating various chronic musculoskeletal pain conditions related to joint, tendon, and nerve pathologies [1]. RF uses a high-frequency alternating current to interrupt or alter nociceptive pathways at various sites, so that it is a useful therapeutic tool to relieve chronic pain syndromes when other conservative or surgical treatments are ineffective or contraindicated, such as in some forms of severe osteoarthritis (OA), for which it is therefore used as a rehabilitation and pain therapy aid [2]. The first clinical use of RF in the treatment of intractable pain was reported in the scientific literature in the early 1970s [3]. It involved the use of conventional currents to create thermal lesions to determine a rhizotomy. In the successive decades and in particular in the last few years, technologies and scientific interest in RF have gradually grown, as the range of musculoskeletal pathologies that represent indications for the use of this pain management treatment has increased considerably.

Two modalities of RF are used in interventional pain medicine. Continuous RF (CRF) is a process whereby the electrical current is used to produce a thermal lesion in a target nerve, resulting in interruption of nociceptive afferent pathways. Pulsed RF (PRF) is a process whereby short bursts of RF are delivered to a target nerve producing effects on signal transduction to reduce pain; this procedure does not produce a neural lesion, but a neuromodulation [4]. A new technique was recently introduced, namely Water-Cooled Radiofrequency (WCRF) [5]. The basic principle of pain relief through WCRF is similar to that of the CRF. However, WCRF provides for the application of a specialized multichannel needle electrode that is actively cooled by the continuous flow of water at ambient temperature, producing a neural lesion in a target area that is wider but better delimited than that created by CRF. Regardless of the type of RF, this promising percutaneous technique aims at relieving chronic pain, even if its mechanisms of neurophysiological functioning remain partly unknown. Despite the ever-increasing clinical application of RF as an interventional procedure to treat musculoskeletal pain, especially that related to OA, a recent systematic review and meta-analysis has only investigated the use of radiofrequency for painful knee OA [6]. To the best of our knowledge, solid evidence is still needed on the efficacy of this therapy, as studies on this subject are limited in terms of sample size, timing, and methodologies for monitoring clinical results.

Therefore, by the present systematic review and meta-analysis, we aimed to evaluate the efficacy of RF in treating chronic musculoskeletal pain, deepening each of the main anatomical areas to which they are usually applied.

## 2. Materials and Methods

### 2.1. Search Strategy

PubMed, Medline, Cochrane, and Pedro databases were systematically searched for English-language articles, according to each specific thesaurus. The following keywords were used in the research: “radiofrequency AND radicular pain” OR “radiofrequency AND chronic pain” OR “radiofrequency AND musculoskeletal pain” OR “radiofrequency AND neuro-modulation” or “radiofrequency AND percutaneous”. This systematic review with meta-analysis was conducted according to the guidance of Preferred Reporting Items for Systematic Reviews and Meta-Analyses (PRISMA) guidelines.

### 2.2. Eligibility Criteria

All RCTs were assessed for eligibility according to the following patient/population, intervention, comparison, and outcomes (PICO) model:

- Participants: patients with chronic musculoskeletal pain, aged between 18 and 80;
- Interventions: CRF, PRF, WCRF;
- Comparison: placebo, anesthetic injection, corticosteroid injection, prolotherapy, conservative treatment (physiotherapy) and transcutaneous electrical nerve stimulation (TENS);
- Outcomes: RF effects on pain and motor disability.

Two reviewers independently screened all potential articles for eligibility after duplication removal. Any disagreement was resolved through discussion or, if necessary, by consultation of a third reviewer. Exclusion criteria were as follows: (1) studies performed on animals; (2) studies about cancer pain or about other types of pain other than musculoskeletal pain; PEDro scale ≤ 5; (3) cross-over study design; (4) studies written in a language other than English; and (5) full-text unavailability (i.e., posters and conference abstracts).

### 2.3. Data Extraction

Two reviewers independently extracted data from the included studies using customized data extraction on a Microsoft Excel sheet. In cases of disagreement, consensus was achieved through a third reviewer. For each study, the following data were collected: first author; year of publication; number of patients; study interval; follow-up duration; baseline characteristics; radiofrequency setting parameters; control-group treatments; types of radiofrequency; treatment target; anatomical site of pain; pain and disability scales.

### 2.4. Data Synthesis

The papers were synthesized describing extracted data. Analysis of the scientific and methodological quality of the studies was carried out using the PEDro Scale. The PEDro scale [7] was developed in 1999 to evaluate the risk of bias and the completeness of statistical reporting of trial reports indexed in the PEDro evidence resource and is now largely used in systematic reviews. This scale evaluates 11 items: inclusion criteria and source, random allocation, concealed allocation, similarity at baseline, subject blinding, therapist blinding, assessor blinding, completeness of follow up, intention-to-treat analysis, between-group statistical comparisons, and point measures and variability. Each item is rated as “yes” or “no”, and the total PEDro score is the number of satisfied items (excluding inclusion criteria and source item). Eight items evaluate risk of bias (random allocation, concealed allocation, similarity at baseline, subject blinding, therapist blinding, assessor blinding, completeness of follow up, intention-to-treat analysis), and two items evaluate the completeness of statistical reporting (between-group statistical comparisons, and point measures and variability). The evaluation of the clinimetric properties of the PEDro scale reveals acceptable validity and reliability. We selected only studies matching as inclusion criteria a PEDRO score > 6, and we excluded studies about hemicrania and peripheral neuropathy.

### 2.5. Statistical Analysis

The continuous variables were extracted and analyzed as mean and standard deviation (SD) using an Excel spreadsheet (Microsoft Corporation, Redmond, WA, USA). If it was not possible to calculate the SD from the available data, the highest SD was used. The standardized mean difference (SMD) and 95% confidence interval (CI) were calculated for continuous variables referring to the same anatomical region and clinical outcome. Higgins’ I2 statistics was calculated to determine the heterogeneity. The pooled estimates of the effect size were presented as forest plots for each condition. The Mantel–Haenszel random-effects model was used to pool the data if statistically significant heterogeneity was reached; the fixed-effects model was used otherwise. Statistical significance was set at *p* < 0.05. All the analyses were conducted in MedCalc Statistical Software version 19.2.6 (MedCalc Software Ltd., Ostend, Belgium).

## 3. Results

**Figure 1:**
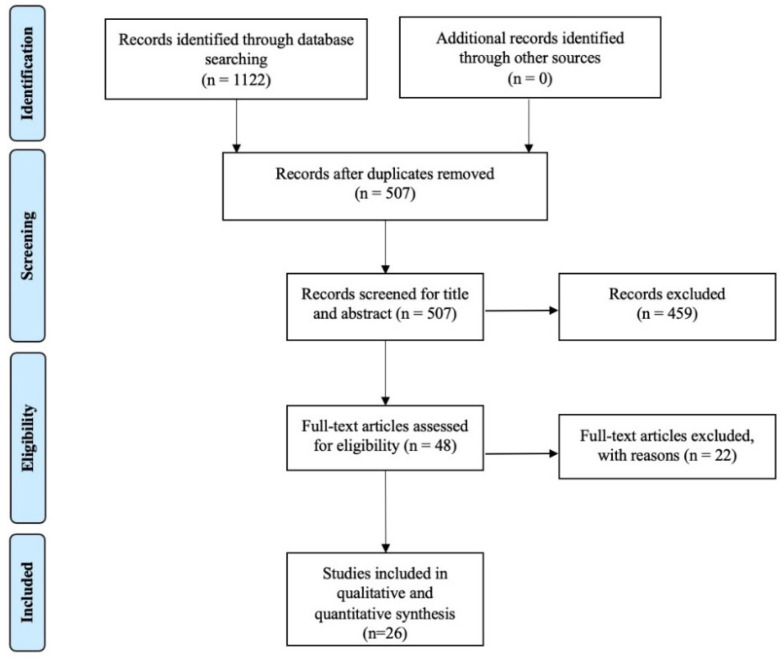
PRISMA flow chart.

A total of 1122 articles was found in all searches in the databases through the applied research strategy. After removing the duplicates, 507 papers were reviewed and filtered by relevance in terms of title and abstract, thus excluding 459 articles. Thus, 48 full-text articles were identified and retrieved for a detailed evaluation. Therefore, 26 RCTs were included in our systematic review (see Figure 1 for PRISMA flow diagram).

Specifically, the following studies were selected: 15 RCTs [8,9,10,11,12,13,14,15,16,17,18,19,20,21,22] regarding the spine RF; 5 RCTs [23,24,25,26,27] about the knee RF; 3 RCTs [28,29,30] about the SI joint RF, and 3 RCTs [31,32,33] regarding the shoulder RF. The total sample size consisted of 1416 patients, both female and male. The average age could not be specified since not all the studies reported this parameter. The characteristics of these items are shown in Table 1.

The efficacy of this procedure was assessed through specific rating outcomes:

- To evaluate pain relief, the following scales were considered: Visual Analogical Scale (VAS) and Numeric Rating Scale (NRS);
- To evaluate improvement in motor disability and articular functionality, the following scales were considered: Oswestry Disability Index (ODI); Oxford Knee score; the physical component of Short Form-36 (SF-36); Western Ontario and McMaster University (WOMAC) Osteoarthritis Index; Shoulder Pain and Disability Index (SPADI) questionnaire.

The complete results are presented in Table 2. Medium and large effects in favor of the control group (SMD < 0) were found for the shoulder VAS and SI Oswestry outcomes, respectively. A small effect in favor of the intervention group (SMD > 0) was found for the spine SF36 outcome. Non-significant SMD was found for the other outcome parameters.

**Figure 2:**
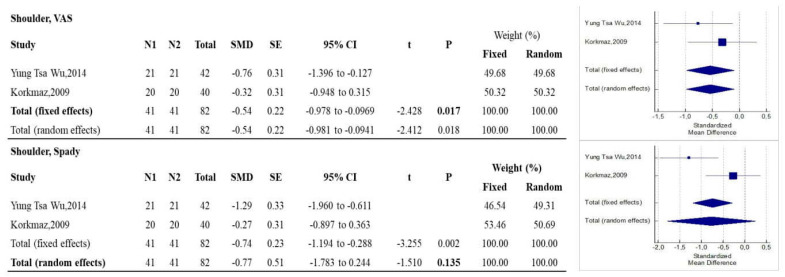
Forest plots showing the intervention effect on the shoulder VAS and Spady outcomes. The use of fixed or random effects (bold) is chosen based on the results of the Higgins’ heterogeneity test. Note: N1 = no. of patients in the intervention group; N2 = no. of patients in the control group; SMD = standardized mean difference; CI = confidence interval.

As reported in Figure 2, RF was more effective than control treatments in reducing pain according to VAS measurements (*p* = 0.017), while there were no statistically significant differences with regard to function measured using SPADI (*p* = 0.035).

**Figure 3:**
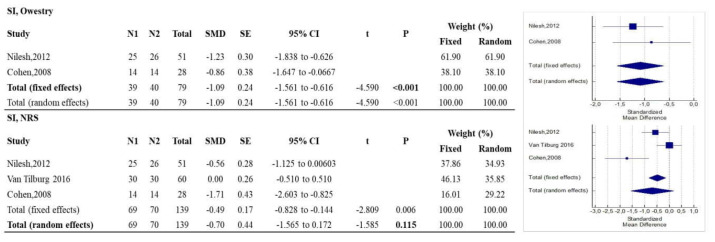
Forest plots showing intervention effects on the sacroiliac Owestry and NRS outcomes. The use of fixed or random effects (bold) is chosen based on the results of the Higgins’ heterogeneity test. Note: N1 = no. of patients in the intervention group; N2 = no. of patients in the control group; SMD = standardized mean difference; CI = confidence interval.

Regarding sacroiliac joint pain, RF was more effective than sham controls in improving articular functionality (*p* < 0.001) according to ODI, while there were no statistically significant differences with regard to pain relief, measured by NRS (*p* = 0.115), as described in Figure 3.

**Figure 4:**
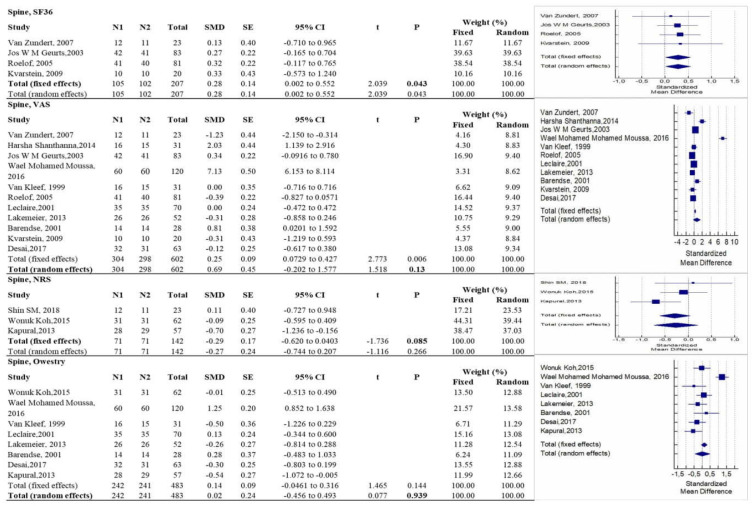
Forest plots showing intervention effects on the spineSF36, VAS, NRS, and Owestry outcomes. The use of fixed or random effects (bold) is chosen based on the results of the Higgins’ heterogeneity test. Note: N1 = no. of patients in the intervention group; N2 = no. of patients in the control group; SMD = standardized mean difference; CI = confidence interval.

As described in Figure 4, RF seemed more effective than control treatments in improving quality of life according to SF-36 in patients suffering from neck pain and low back pain (*p* = 0.043), while there were no statistically significant differences with regard to pain relief, according to VAS and NRS, and to function, according to ODI. However, these data must also be read in light of the statistical methodology we have used, exactly as for the sacroiliac and shoulder anatomical regions. In fact, if we observe, for example, the results related to the spine VAS (Figure 4), it is possible to note how the RF is effective in relation to the value of the fixed effect (*p* = 0.006), but this improvement cannot be considered consistent and homogeneous in the comparison between all the studies considered. Therefore, we must consider the random effect, which, by virtue of these data, is not sufficient to affirm the superiority of one treatment over another (*p* = 0.13).

**Figure 5:**
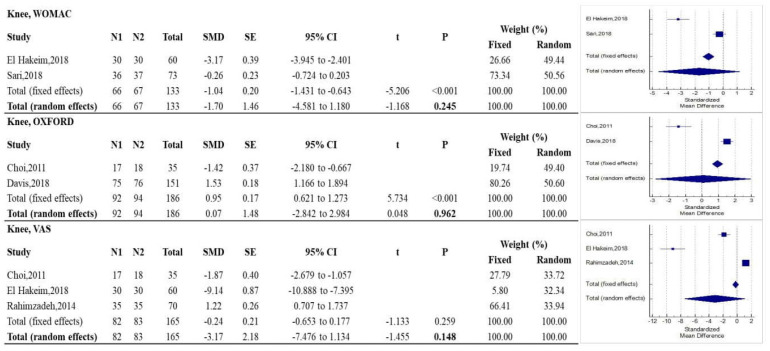
Forest plots showing intervention effects on the knee: WOMAC, OXFORD, and VAS outcomes. The use of fixed or random effects (bold) is chosen based on the results of the Higgins’ heterogeneity test. Note: N1 = no. of patients in the intervention group; N2 = no. of patients in the control group; SMD = standardized mean difference; CI = confidence interval.

As described in Figure 5, there were no statistically significant differences between RF and control treatments on pain relief, according to VAS, and on function, according to WOMAC and Oxford Knee scales.

## 4. Discussion

Musculoskeletal pain has a great impact on patients’ quality of life, considering that it might determine sleep interruption, fatigue, depressed mood, activity limitations, and participation restrictions, and it is even more disabling when the pain is related to sport or work [34,35,36,37,38,39,40,41]. The diagnosis itself is often affected by cultural and psychological factors that make it difficult to correctly identify the cause of chronic musculoskeletal pain [42]. Moreover, joint pain, although justified by OA, retains mixed characteristics due to the complexity of this disease, which involves multiple tissues and determines various clinical syndromes. In fact, although central nociceptive pathways contribute to OA pain, crosstalk between the immune system and nociceptive neurons is central to this pain; therefore, new therapies and new diagnostic tools might target this crosstalk [43]. Traditionally, the treatment of chronic musculoskeletal pain is based on a multidisciplinary approach, which aims to use different therapies optimizing the results and limiting the possible side effects. Rehabilitation and analgesic or anti-inflammatory drugs are undoubtedly safe and effective treatments, albeit they are not always sufficient to achieve satisfactory pain relief [44,45,46]; therefore, in these cases it may be useful to use minimally invasive therapies such as RF. By the present systematic review and meta-analysis, we investigated the RF effectiveness on different types of musculoskeletal pain, distinguishing it according to the different anatomical regions of application.

### 4.1. Shoulder

With regard to the shoulder, the studies we selected deepened the efficacy of RF on adhesive capsulitis, showing that RF was more effective than control treatments in reducing pain (*p* = 0.017). A recent meta-analysis [47] confirmed these results: from an analysis of seven selected trials they found that pulsed RF for chronic shoulder pain provided similar analgesia as conservative medical management at three months after the procedures. On the contrary, the authors found that RF seems to be effective from a functional point of view: these data appear in contrast with our results but could be explained by the fact that the only diagnosis considered in our study was that of adhesive capsulitis, while Pushparaj et al. included in their study also other diagnosis, such as osteoarthritis and rotator cuff tears, in which the beneficial effects of RF on pain were translated more immediately into motor and functional advantages. Moreover, new studies are also providing new evidence on RF for treating frozen shoulder. Yan et al. [48] demonstrated that a group of 68 patients treated by ultrasound-guided pulsed RF achieved significant improvement in both pain and function measured with SPADI and quality of life measured with SF-36 up to 12 weeks of follow-up in comparison with a sham group. These data seem to be confirmed also by Ergonenc et al. [49], thus suggesting the usefulness of RF in treating this disease but also the importance of performing it in an ultrasound-guided manner.

### 4.2. Sacroiliac Joint

Regarding the SI, an interesting meta-analysis by Chen et al. [50] confirmed our results regarding the ODI improvement, but it apparently disagreed with our results concerning pain. A possible explanation of this difference lies in the fact that, as the authors declared, the studies they selected were heterogeneous for measurement scales and control groups with reference to pain. To this it should be added that the origin of sacroiliac joint pain is still controversial, since in some cases, up to 30%, it can derive from dysfunctions and pathologies of the lumbar spine [51]. Nevertheless, RF seems to be a precious option for sacroiliac chronic pain, especially when other conservative therapies are ineffective and above all with a view to implementing the joint function in the context of integrated rehabilitation programs [52].

### 4.3. Spine

About the spine, our findings are in line with a Cochrane collaboration’s systematic review [53], which affirmed that there is limited evidence that RF denervation offers short-term relief for chronic neck pain of zygapophysial joint origin and for chronic cervicobrachial pain, and conflicting evidence for its effectiveness for lumbar zygapophysial joint pain, since further high-quality randomized controlled trials are needed. Nevertheless, the most recent literature updates, also in terms of meta-analyses, seem encouraging: conventional RF denervation resulted in reductions in low back pain originating from the facet joints in patients showing the best response to diagnostic block over the first 12 months when compared with sham procedures or epidural nerve blocks [54]. Moreover, RF seems effective for alleviating cervical radicular pain, when it is unresponsive to oral medications, physical therapy, or epidural steroid injection [55]. Among the different RF types, the WCRF is proving to be as or more effective than previous RF technologies in terms of pain reduction and implementation of joint function in the lumbar spine [56]. Finally, a systematic review by Manchikanti et al. [57] placed at a level II of evidence the lumbar and cervical RF neurotomy as a long-term improvement tool for pain, joint function, and quality of life in patients suffering from spine pain.

### 4.4. Knee

The knee is traditionally the most investigated anatomical region, not only to understand the effectiveness of RF but also to deepen its diagnostic accuracy and functioning mechanism [58]. Genicular blockade and RF ablation are effective diagnostic tools ex adiuvanitbus, since this combination allows the cause of pain to be identified with the utmost precision and to execute a targeted therapy at the same time [59].

With regard to the knee, we did not find any statistically significant difference between RF and control treatments on pain relief, according to VAS, and on function, according to WOMAC and Oxford Knee scales. In this case, it is obviously necessary to make a consideration such as the one reported above in reference to spine pain. That is, by sticking to the statistical tools used, it is not possible to affirm the superiority of RF over other treatments due to the heterogeneity of the selected studies. Nevertheless, the available literature is very encouraging about the use of RF in knee osteoarthritis, which represents a good pathological model for RF indications. In fact, when knee osteoarthritis is severe, it causes a chronic pain that is often disabling and poorly responsive to traditional therapies. In some cases, this disease cannot be resolved by surgery, as it is not practicable by virtue of comorbidities that prevent it; even drugs may be limited in results due to addiction or excessive side effects. RF is thus a valid option to contrast knee chronic pain. In a recent review by Airawat et al., RF improved pain, functionality, and quality of life for up to three to twelve months with minimal localized complications for patients with knee OA who were unresponsive to conservative therapies [60]. These findings were confirmed by Chen et al. [61], who demonstrated that geniculate nerve thermal RF is a superior nonsurgical treatment of knee OA compared with non-steroidal anti-inflammatory drugs (NSAIDs) and intra-articular corticosteroid injections. Moreover, knee RF are now carried out more and more frequently with ultrasound guidance, which is as effective as fluoroscopic guidance, but easier to apply, as well as free from ionizing radiation [6].

### 4.5. Radiofrequency: An Opportunity for Musculoskeletal Rehabilitation and Pain Medicine

To our knowledge, this is the first systematic review and meta-analysis that evaluates the efficacy of RF on the chronic MP deepening of each of the main anatomical areas to which they are usually applied. RF is a promising pain therapy, especially when other treatments are ineffective. Since it is firstly and foremost an analgesic therapy, it could be included in global rehabilitation projects, in order to obtain better results from a functional point of view: the association with other rehabilitation treatments can enhance the RF benefits [2,62]. In fact, the results of RF are long-lasting but transient in relieving pain, since injured nerve branches tend to regenerate progressively due to the sprouting phenomenon starting from the basal lamina of Schwann cells [63]. Therefore, the pain reduction window is an opportunity for rehabilitation to recover joint function and implement muscle trophism, triggering a virtuous cycle of maintaining the well-being of the musculoskeletal system. As partially announced, all the results described are affected by a vulnerability in the scientific literature currently existing on RF. In fact, high-quality trials are still needed, with larger patient samples and data on long-term effects. Moreover, the most used outcome measures are inherently weak in their ability to objectify results referred to pain and function; thus, a limited number of studies with different outcomes and body region evaluated were included in the study. Direct comparison of the study outcomes was also limited by their heterogeneity. Finally, setting up a case-control study is always complex when minimally invasive therapies are used, both from an ethical point of view and from the point of view of clinical practice and experience. Nevertheless, these results give hope that RF constitutes a good therapeutic perspective for treating MP and that it can be supported by new solid evidence based on emerging quality clinical studies.

## 5. Conclusions

This systematic review and meta-analysis showed that RF might represent a promising therapy for the treatment of chronic musculoskeletal pain, especially when other approaches are ineffective or not practicable. Further studies are needed to clarify the effectiveness of RF on pain and joint function for each anatomical region of common application.

## Figures and Tables

**Table 1:** Main characteristics of the studies included.

Study	Year	PEDro Score	Sample Size	Anatomical Region of Musculoskeletal Pain	Type of Radiofrequency	Target	Outcome	Control Group	Radiofrequency Method
Min Shin et al. [8]	2018	8	23	Chronic suboccipital neck pain	Pulsed	Occipital-atlas Joint	NRS	Intra-articular Corticosteroid	42° × 360 s
Zundert et al. [9]	2007	9	23	Neck pain	Pulsed	Medial branch of the dorsal ramus	VAS, GPE, SF-36	Sham without RF	NS × 120 s
Roelof et al. [10]	2005	9	81	Low back pain	Pulsed	Medial branch of the dorsal ramus	VAS, SF-36	Sham without RF	80° × 60 s
Sherdil et al. [11]	2008	9	40	Low back pain	Pulsed	Medial branch of the dorsal ramus	VAS, ROM	Sham without RF	85° × 60 s
Leclaire et al. [12]	2001	7	70	Low back pain	Pulsed	Medial branch of the dorsal ramus	VAS, ODI, RMDQ	Sham without RF	80° × 90 s
Lakemeier et al. [13]	2013	8	52	Low back pain	Continue	Medial branch of the dorsal ramus	VAS, ODI, RMDQ	Intra-articular Corticosteroid	80° × 90 s
Moussa and Khedr [14]	2016	8	120	Low back pain	Continue	Medial branch of the dorsal ramus Facet joint capsule	VAS, ODI	Sham without RF	85° × 90 s
Geurts et al. [15]	2003	10	83	Low back pain	Continue	Dorsal root ganglion	VAS, DAS, SF-36, MRI findings, NARS	Sham without RF	67° × 90 s
Shanthanna et al. [16]	2014	10	31	Low back pain	Pulsed	Dorsal root ganglion	VAS, ODI	Sham without RF	42° × 120 s
Koh et al. [17]	2015	10	62	Low back pain	Pulsed	Dorsal root ganglion	NRS, ODI, MQS, GPE	Epidural	42° × 120 s
Van Kleef et al. [18]	1999	10	31	Low back pain	Continue	Medial branch of the dorsal ramus	VAS, ODI, CWQS	Sham without RF	80° × 60 s
Kvarstein et al. [19]	2009	10	20	Low back pain	Continue	Intradiscal space	NRS, ODI, SF-36	Sham without RF	65° × 10 min
Barendse et al. [20]	2011	9	28	Low back pain	Continue	Intradiscal space	VAS, ODI, CWQS	Sham without RF	70° × 90 s
Desai et al. [21]	2017	7	63	Low back pain	Cooled	Intradiscal space	VAS, ODI, SF-36, EQ5D-VAS, EQ5D-HI, PGIC, BDI	Kinesitherapy	NS
Kapural et al. [22]	2013	9	57	Low back pain	Cooled	Intradiscal space	NRS, ODI, SF-36	Sham without RF	45 °C bipolar for 15 min, then 50 °C in bipolar for 15 min, then 60 °C monopolar for 2.5 min
Choi et al. [23]	2011	9	35	Knee osteoarthritic pain	Continue	Geniculate nerves	VAS, OKS, GPE	Sham without RF	70° × 90 s
El-Hakeim et al. [24]	2018	7	60	Knee osteoarthritic pain	Continue	Geniculate nerves	VAS, WOMAC-OI	Paracetamol and diclofenac per os	80° × 90 s 3 cycles
Sari et al. [25]	2018	7	73	Knee osteoarthritic pain	Continue	Geniculate nerves	VAS, WOMAC-OI	Infiltration with betamethasone and morphine	80° × 90 s
Davis et al. [26]	2018	6	151	Knee osteoarthritic pain	Cooled	Geniculate nerves	NRS, OKS	Corticosteroid infiltration	60° × 150 s
Rahimzadeh et al. [27]	2014	9	70	Knee osteoarthritic pain	Pulsed	Geniculate nerves	VAS, Knee ROM, GPE	Erythropoietin infiltration	42° × 15 min 2 cycles
Van Tilburg et al. [28]	2016	9	60	Chronic sacroiliac pain	Cooled	Lateral branch of the dorsal ramus	NRS, GPE	Sham without RF	85° × 90 s
Cohen et al. [29]	2008	8	28	Chronic sacroiliac Pain	Cooled	Lateral branch of the dorsal ramus	NRS, GPE, ODI	Sham without RF	80° × 90 s
Patel et al. [30]	2012	9	51	Chronic sacroiliac pain	Cooled	Lateral branch of the dorsal ramus	NRS, SF-36, ODI, AQoL	Sham without RF	60° × 150 s
Wu et al. [31]	2014	7	42	Shoulder adhesive capsulitis	Pulsed	Suprascapular nerve	SPADI, ROM, VAS	Kinesitherapy	42° × 180 s
Korkmaz et al. [32]	2009	7	40	Shoulder adhesive capsulitis	Pulsed	Suprascapular nerve	VAS, ROM, SF-36, SPADI	TENS	42° × 360 s
Gofeld et al. [33]	2012	8	22	Shoulder adhesive capsulitis	Pulsed	Suprascapular nerve	NRS, SPADI, CMS	Sham without RF	42° × 120 s

Abbreviations: NRS, Numeric Rating Scale; VAS, Visual Analogical Scale; GPE, Global Perceived Effect; SF-36, Medical Outcomes Study 36-Item Short-Form Health Survey; ROM, Range of Motion; ODI, Oswestry Disability Index; RMDQ, Roland and Morris Disability Questionnaire; DAS, daily activities scale; MRI, magnetic resonance imaging; NARS, numerical analgesics rating scale, MQS Medication Quantification Scale; CWQS, Coop-wonka quality scale; EQ5D-HI, EuroQol 5 dimensions Health index; PGIC, Patient Global Impression of Change; BDI, Beck Depression Inventory; OKS, Oxford Knee Score; WOMAC-OI Western Ontario and McMaster University Osteoarthritis index; AQoL Assessment of Quality of Life; SPADI Shoulder Pain and Disability Index; CMS, Constant–Murley Scale; NS, not specified; TENS, Transcutaneous Electrical Nerve Stimulation; s, seconds; min, minutes.

**Table 2:** Summary on the outcome measures according to body district.

Body Region	Outcome	Study (First Author, Year)	Intervention Group			Control Group		
			N	Mean	SD	N	Mean	SD
Knee	OXFORD KNEE SCORE	Choi, 2011	17	27.4	10.2	18	38.9	4.8
		Davis, 2018	75	35.7	8.8	76	22.4	8.5
	VAS	Choi, 2011	17	4.2	2.5	18	7.8	1.0
		El Hakeim, 2018	30	3.1	0.3	30	5.7	0.3
		Sari, 2018	36	4.0	-	37	5.5	-
		Davis, 2018	75	2.5	-	76	6.0	-
		Rahimzadeh, 2014	35	5.5	1.9	35	3.5	1.2
	WOMAC	El Hakeim, 2018	30	33.1	4.1	30	43.5	2.0
		Sari, 2018	36	39.7	8.9	37	42.3	11.0
Sacroiliac	NRS	Nilesh, 2012	25	3.6	2.6	26	5.0	2.4
		Van Tilburg 2016	30	5.4	1.7	30	5.4	1.9
		Cohen, 2008	14	2.4	2.0	14	6.3	2.4
	OSWESTRY	Nilesh, 2012	25	24.0	16.0	26	39.0	6.0
		Cohen, 2008	14	33.3	10.6	14	42.1	9.3
Shoulder	SPADI DISABILITY	Yung Tsa Wu, 2014	21	15.0	12.3	21	35.2	18.0
		Korkmaz, 2009	20	9.9	7.9	20	12.4	10.3
		Gofeld, 2012	11	35.2	-	11	45.5	-
	VAS	Yung Tsa Wu, 2014	21	1.7	1.5	21	3.3	2.5
		Korkmaz, 2009	20	1.8	0.9	20	2.1	1.0
Spine	NRS	Shin SM, 2018	12	2.8	1.7	11	2.6	1.8
		Wonuk Koh, 2015	31	5.7	4.9	31	6.2	5.5
		Kapural, 2013	28	4.9	2.4	29	6.5	2.1
	OSWESTRY	Wonuk Koh, 2015	31	37.6	32.7	31	38.0	32.5
		Harsha Shanthanna, 2014	16	40.2	0.2	15	4.9	0.1
		Wael Mohamed Moussa, 2016	60	33.9	31.6	60	5.9	0.9
		Van Kleef, 1999	16	31.0	14.2	15	38.0	13.1
		Leclaire, 2001	35	38.3	14.7	35	36.4	14.6
		Lakemeier, 2013	26	28.0	20.0	26	33.0	17.4
		Barendse, 2001	14	43.7	11.6	14	40.7	9.5
		Desai, 2017	32	22.0	28.0	31	29.0	16.0
		Kapural, 2013	28	32.9	16.1	29	41.2	13.9
	SF36	Van Zundert, 2007	12	9.0	16.6	11	6.9	15.0
		Jos W M Geurts, 2003	42	40.0	15.7	41	36.0	13.6
		Roelof, 2005	41	47.6	16.9	40	41.6	19.7
		Kvarstein, 2009	10	65.0	21.7	10	57.5	21.4
	VAS	Van Zundert, 2007	12	5.6	1.7	11	7.6	1.4
		Harsha Shanthanna, 2014	16	6.8	3.2	15	1.5	1.6
		Jos W M Geurts, 2003	42	5.2	2.2	41	4.4	2.4
		Wael Mohamed Moussa, 2016	60	6.0	1.0	60	0.7	0.3
		Van Kleef, 1999	16	5.2	1.7	15	5.2	1.6
		Roelof, 2005	41	5.8	1.8	40	6.5	1.8
		Leclaire, 2001	35	5.2	26.7	35	5.2	20.8
		Lakemeier, 2013	26	4.7	2.4	26	5.4	2.1
		Nath, 2008	20	3.9	-	20	3.7	-
		Barendse, 2001	14	6.5	1.3	14	5.5	1.1
		Kvarstein, 2009	10	3.6	2.6	10	4.5	2.9
		Desai, 2017	32	4.4	2.9	31	4.7	2.0

## Data Availability

The datasets used and/or analyzed during the current study will be made available upon reasonable request to the corresponding author, G.F.
